# Salivary concentrations of macrophage activation-related chemokines are influenced by non-surgical periodontal treatment: a 12-week follow-up study

**DOI:** 10.1080/20002297.2019.1694383

**Published:** 2019-12-09

**Authors:** Maria A. Grande, Daniel Belstrøm, Christian Damgaard, Palle Holmstrup, Eija Könönen, Mervi Gursoy, Ulvi Kahraman Gursoy

**Affiliations:** a Section for Periodontology, Microbiology and Community Dentistry, Department of Odontology, Faculty of Health and Medical Sciences, University of Copenhagen, Copenhagen, Denmark; b Department of Cancer and Inflammation, Institute of Molecular Medicine, Faculty of Health and Medical Sciences, University of Southern Denmark, Odense, Denmark; c Department of Periodontology, Institute of Dentistry, University of Turku, Turku, Finland

**Keywords:** Periodontitis, Saliva, Chemokines, Gram-negative bacteria, Inflammation

## Abstract

**Background:** During periodontal inflammation, bacteria induces chemokine expression and migration of various inflammatory cells. The aim of the study was to learn if periodontal treatment alters salivary concentrations of macrophage activation-related chemokines and if such alterations correlate with abundance of periodontitis-associated bacteria.

**Methods:** Twenty-five patients with periodontitis completed the study (NCT02913248 at clinicaltrials.gov). Periodontal parameters and stimulated saliva samples were obtained at baseline and 2, 6 and 12 weeks after non-surgical periodontal treatment. Salivary concentrations of monocyte chemoattractant proteins (MCP-1-4), macrophage-derived chemokine (MDC), macrophage migration inhibitory factor (MIF), monokine induced by interferon-gamma (MIG), macrophage inflammatory protein (MIP-1α) and interferon-inducible protein (IP-10) were quantified using the Luminex® xMAP™ technique and abundance of bacteria was quantified using next-generation sequencing.

**Results:** The treatment improved all periodontal parameters and caused an increase in the concentrations of MCP-2, MDC and MIP-1α at week 12 compared to baseline, week 2 and week 6, respectively. Salivary concentrations of MCP-1-2, MDC, MIG, MIP-1α and IP-10 correlated with the abundance of specific periodontitis-associated bacteria.

**Conclusions:** Periodontal treatment impacts salivary concentrations of MCP-2, MDC and MIP-1α, which correlate with the abundance of specific periodontitis-associated bacteria. This indicates that these chemokines reflect periodontal status and possess potential in illustrating a response to treatment.

## Introduction

Chemokines are referred to as ‘chemotactic cytokines’ as they activate and promote migration of a variety of inflammatory cells by dictating the type and magnitude of immune response [1]. A persistent production of chemokines and recruitment of leukocytes characterizes chronic inflammation such as periodontitis [2,3].

Periodontitis is a multifactorial inflammatory disease affecting up to 50% of the adult population in the Western world [4]. Etiologically, a polymicrobial biofilm initiates and maintains a destructive inflammatory response in the tooth-supporting tissues. Specifically, subgingival presence of the red complex bacteria *Porphyromonas gingivalis, Treponema denticola* and *Tannerella forsythia* strongly associate with periodontitis [5]. Lipopolysaccharide (LPS), a biologically active structure on the Gram-negative bacteria, can be recognized by pattern recognition receptors (PRRs) and activate resident cells to produce several chemokines, e.g. interleukin (IL) −8 (CXCL8), monocyte chemoattractant proteins (MCPs), macrophage inflammatory protein (MIP)-1α and interferon-inducible protein (IP)-10 [6]. Increased concentrations of MCP-1, MCP-3, macrophage migration inhibitory factor (MIF), MIP-1α, IP-10 and macrophages have been demonstrated in inflamed gingival biopsies, as compared to biopsies from healthy gingival sites [7–11]. Moreover, increased concentrations of chemokines like MIP-1α in saliva have been reported to associate with periodontitis [12–16]. This suggests that concentrations of chemokines in saliva can reflect local periodontal inflammation.

Cross-sectional studies have demonstrated an increase in salivary concentrations of both microbial- and host-inflammatory biomarkers in patients with periodontitis [14,17–20]. However, longitudinal studies, which test saliva’s ability to reflect periodontal inflammation are limited [16,21–23]. In a recently performed longitudinal study, we found, that saliva reflects subgingival abundance of specific bacteria associated with periodontitis; *P. gingivalis, T. denticola, T. forsythia, Prevotella intermedia, Parvimonas micra* and *Filifactor alocis* before and after non-surgical periodontal treatment [24]. The aim of the present study was, therefore, to use the same salivary samples to learn if periodontal treatment influences the macrophage activation-related chemokine concentrations in saliva. We tested the hypotheses that non-surgical periodontal treatment has impact on salivary concentrations of the chemokines; MCP-1(CCL2), MCP-2(CCL8), MCP-3(CCL7), MCP-4(CCL13), macrophage-derived chemokine (MDC or CCL22), MIF, monokine induced by interferon-gamma (MIG or CXCL9), MIP-1α and IP-10 (CXCL10). Thereto, that changes in salivary chemokine concentrations will correlate with salivary abundance of *P. gingivalis, T. denticola, T. forsythia, P. intermedia, P. micra* and *F. alocis.*

## Materials and methods

### Study population

This study is part of a series of studies of a population, which has previously been described in detail [24]. Briefly, 31 patients with moderate to severe generalized periodontitis, as defined by the American Academy of Periodontology [25], were enrolled in the study. All patients approved their study participation by signing a written informed consent before commencement. Inclusion criteria: Age ≥ 40 yrs., teeth ≥ 20, Caucasian. Exclusion criteria: Treatment involving caries, hyposalivation, systemic diseases, current use of medication with known effect on the periodontium, professional dental cleaning or/and antibiotic treatment within the last 3 months. The study was accepted by the regional ethical committee of the capital region of Denmark (H-16,016,368), reported to the Danish Data Authority (SUND-2016-58) and registered at clinicaltrials.gov (NCT02913248).

### Clinical examination and periodontal treatment

The study was performed from September 2016 to the beginning of January 2017 at the Department of Odontology, University of Copenhagen. Non-surgical periodontal treatment was performed at baseline. The treatment consisted of comprehensive individual hygiene instructions followed by scaling and root planing. Full-mouth periodontal recordings (third molars excluded) were obtained at baseline and 12 weeks after therapy. The periodontal recordings were measured at six sites per tooth and included registrations of plaque index (PI), bleeding on probing (BOP), probing pocket depth (PD) and clinical attachment level (CAL). Control visits were performed 2 and 6 weeks after treatment. PI and BOP were recorded, and oral hygiene instructions were repeated, if plaque was present upon application of erythrosine. All recordings and treatments were performed by the same clinician (MAG).

### Saliva sampling

Stimulated saliva samples were collected at baseline and 2, 6 and 12 weeks after the non-surgical periodontal treatment. Samples were collected from 8 am to 3 pm by the same clinician (MAG). Sampling was performed from each patient at each visit within a time-interval of 4 h. Before sampling, the participants flushed their mouth with tap water, and thereafter paraffin-stimulated whole saliva was collected to a minimum of 2 mL. All samples were immediately frozen, then stored at −80°C until further analysis at the University of Turku laboratories.

### Analysis of salivary chemokines

Saliva samples were thawed and centrifuged at 9300 *g* for 5 min at room temperature. MCP-1, MCP-2, MCP-3, MCP-4, macrophage-derived chemokine (MDC), MIF, monokine induced by IFN-gamma (MIG), MIP-1α and IP-10 were identified in supernatants using the Luminex xMAP technic (Luminex Corporation, Austin, TX) with optimized commercial kits (Pro-human cytokine group 1 assays; Bio-Rad, Santa Rosa, CA), according to the manufacturers’ protocol.

The limit of detection (LOD) for the assay, i.e. the minimum concentration of an analyte where the fluorescence intensity signal could be detected, was 0.1 pg/ml for MCP-1, 0.04 pg/ml for MCP-2, 1.3 pg/ml for MCP-3, 0.1 pg/ml for MCP-4, 0.5 pg/ml for MDC, 15.4 pg/ml for MIF, 1.1 pg/ml for MIG, 0.3 pg/ml for MIP-1α and 1.1 pg/mg for IP-10.

### Analysis of bacteria

DNA isolation and next-generation sequencing were performed as previously described in detail [24]. In brief, bacterial DNA was isolated using MagNA Pure 96 instrument (Roche, Mannheim, Germany). Next-generation sequencing of the V3-V4 region of the 16S rDNA gene was performed using the Human Oral Microbe Identification Using Next Generation Sequencing (HOMI*NGS*) technique. Sequences were blasted against reference sequences in Probeseq, which contains 692 reference sequences based on the HOMD database [26].

### Statistics

All data were checked for normal distribution with box-plot- and histogram-illustrations. Repeated t-test was used to compare the clinical data (PI, BOP, PD, CAL). Friedman’s test was used to analyze changes in chemokine concentrations (p < 0.01). When significant alterations were observed Wilcoxon signed-rank test with Bonferroni correction was used as post hoc analysis. By applying the Bonferroni correction, a p-value <0.008 was computed and considered as statistically significant. Correlations of chemokine concentrations with other chemokines and abundance of specific bacteria associated with periodontitis were tested using Spearman’s signed-rank test (p < 0.05). All statistical analyses were performed in Graph Pad Prism v8 (Graph Pad Software, San Diego, California).

## Results

The study was completed by 25 patients [24]. One tube of saliva broke during centrifugation, which resulted in exclusion of all samples from this patient. The chemokine concentrations from the remaining 24 patients were above the detection limit of the test kit, except for MCP-3 concentrations in 10 samples and MCP-2 concentrations in one sample. The MCP-2 and MCP-3 concentrations below the LOD value were substituted with LOD/2 for the statistical analyses [27,28].

### Non-surgical periodontal treatment improved all clinical parameters

The outcome of the clinical parameters has previously been published in detail [24]. In summary, non-surgical periodontal treatment resulted in significant improvements of PI, BOP, PD and CAL throughout the study period (p < 0.001). PI: 84.2% (baseline), 41% (week 2), 43% (week 6), 41,8% (week 12). BOP: 56% (baseline), 27.1% (week 2), 34,6% (week 6), 41.3% (week 12). PD: 3.4 mm (baseline), 3.0 mm (week 12). CAL: 4.1 mm (baseline), 3.7 mm (week 12).

### Effect of non-surgical periodontal treatment on salivary chemokine concentrations

**Figure 1.**
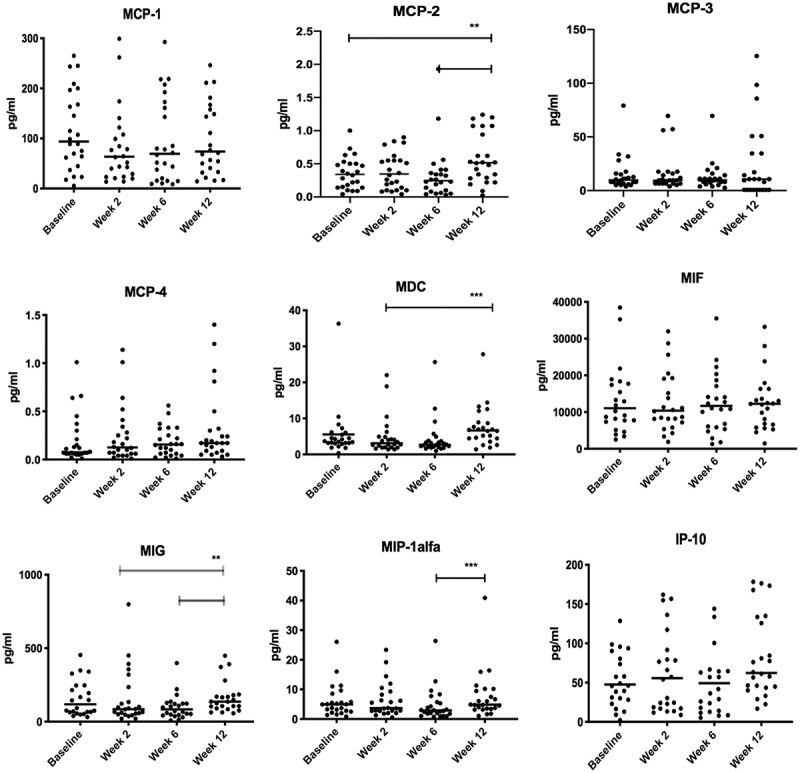
Scatter dot-plot of salivary concentrations of chemokines measured at baseline, week 2, week 6, and week 12. Horizontal-lines:Median-values

Alterations in salivary chemokine concentrations are illustrated in Figure 1. Non-surgical periodontal treatment significantly influenced the median salivary concentrations of MCP-2, MDC, MIG and MIP-1α (p < 0.01). A significant increase in concentrations of MCP-2, MDC and MIP-1α was observed at week 12 compared to baseline, week 2 and 6 (p < 0.008). The salivary concentrations of MCP-1, MCP-3, MCP-4, MIF, and IP-10 did not change significantly during the 12 weeks.

The black bars illustrate significant alterations using the Wilcoxon signed-rank tests with Bonferroni correction (p < 0.008). The faded lines (MIG) illustrate significant alterations, though with a higher P-value than decided by the Bonferroni correction (p < 0.05).

### Correlations of chemokine concentrations

Correlations of salivary chemokine concentrations measured at baseline with concentrations recorded after 2, 6 and 12 weeks are illustrated in Table 1. Significant correlations of the baseline concentrations with those recorded at week 2 were observed for all nine chemokines tested (p < 0.05). At week 6, the concentrations of six chemokines still correlated significantly with baseline concentrations (p < 0.01). However, at week 12 only MCP-1 and MIG concentrations correlated with the baseline values (p < 0.05).

**Table 1.** Correlation coefficients (R) of salivary concentrations of chemokines at baseline with concentrations recorded at week 2, week 6 and week 12. *(*p* < 0.05), **(*p* < 0.01), ***(*p* < 0.001)

Chemokine	Baseline vs week 2	Baseline vs week 6	Baseline vs week 12
MCP-1	0.81***	0.67**	0.65**
MCP-2	0.58**	0.67**	0.22
MCP-3	0.55*	0.51	−0.24
MCP-4	0.50*	0.40	0.075
MDC	0.79***	0.36	0.36
MIF	0.66**	0.55**	0.24
MIG	0.64**	0.54**	0.41*
MIP-1α	0.79***	0.62**	0.39
IP-10	0.67**	0.76***	0.29

When correlations of different chemokine concentrations throughout the study period were tested, significant correlations were observed in selected chemokine groups; MCP-2/MCP-3/MCP-4, MDC/MIG/MIP-1ɑ and MCP-1/MIP-1ɑ/MIG/IP-10 (p < 0.001) (Table 2).

**Table 2.** Correlation coefficients (R) when correlating salivary concentrations of each chemokine at all sampling times *(*p* < 0.05), **(*p* < 0.01), ***(*p* < 0.001), ****(*p* < 0.0001)

	MCP-1	MCP-2	MCP-3	MCP-4	MDC	MIF	MIG	MIP-1alfa	IP-10
MCP-1		0.33**	−0.14	−0.26*	0.41****	0.39****	0.70****	0.62****	0.59****
MCP-2			0.75****	0.40****	0.46***	−0.07	0.31**	0.42****	0.30**
MCP-3				0.73****	0.36**	−0.18	−0.07	0.26*	−0.13
MCP-4					0.24*	−0.13	−0.20	0.07	−0.10
MDC						0.23*	0.50****	0.63***	0.33**
MIF							0.23*	0.41****	0.19
MIG								0.57****	0.69****
MIP-1α									0.49****
IP-10									

### Correlations of salivary chemokine concentrations with abundance of bacteria associated with periodontitis in saliva

Relative abundance of bacteria associated with periodontitis in the saliva samples is previously described [24]. Correlations of salivary abundance of *P. gingivalis, T. denticola, T. forsythia, P. intermedia, P. micra* and *F. alocis* with salivary chemokine concentrations were tested, and data are presented in Table 3. Relative abundance of *P. gingivalis* correlated significantly with MCP-1, MDC and MIP-1ɑ. Correlations of *T. denticola* with MCP-1-2, MIG and IP-10 were also observed. *P. intermedia* correlated with MCP-1 and MIG, *F. alocis* with MCP and MIP-1ɑ and *P. micra* with *IP-10*. No correlations of the abundance of *F. alocis* with salivary concentrations of chemokines existed. (p < 0.05)

**Table 3.** Correlation coefficients (R) of salivary concentrations of chemokines with the salivary relative abundance of six bacteria associated with periodontitis and with the total bacterial burden. The total bacterial burden: The sum of the relative abundance of *P. gingivalis, T. denticola, P. intermedia, F. alocis, T. forsythia* and *P. micra*. *(*p* < 0.05), **(*p* < 0.01)

Chemokine	*P. gingivalis*	*T. denticola*	*P. intermedia*	*F. alocis*	*T. forsythia*	*P. micra*	*Total bacterial burden*
MCP-1	0.21*	0.24*	0.28**	0.20	0.18	−0.08	0.30**
MCP-2	0.09	0.25*	0.14	0.04	−0.01	0.15	0.15
MCP-3	0.15	0.16	−0.08	−0.01	0.05	0.16	0.08
MCP-4	0.19	0.12	−0.15	0.03	0.14	0.20	0.12
MDC	0.28**	0.10	0.01	0.22*	0.03	−0.06	0.11
MIF	0.05	0.19	0.18	0.11	0.14	−0.00	0.24*
MIG	0.09	0.21*	0.27**	0.16	0.01	−0.18	0.09
MIP-1α	0.22*	0.08	0.13	0.36**	0.12	0.02	0.21*
IP-10	0.09	0.24*	0.08	0.11	0.12	−0.27**	0.11

## Discussion

The main finding of the present study was that non-surgical periodontal treatment affects salivary concentrations of MCP-2, MDC, MIG and MIP-1α (Figure 1). Moreover, the observed correlations between tested chemokine indicate that the expression and secretion pathways of these chemokines are inter-related (Table 2). To the best of our knowledge, this study is the first to analyze salivary concentrations of MCP-2, −4, MDC, MIG and IP-10 in periodontitis patients with a longitudinal design.

In general, the effect of non-surgical periodontal treatment on salivary concentrations of macrophage activation-related chemokines has only been tested in a limited number of studies [16,21]. In line with data from the present study, steady salivary MIP-1α concentrations have been reported in samples collected at baseline and 16 weeks after periodontal therapy [21]. In contrast, significant decreases in MCP-1 concentrations in saliva, gingival crevicular fluid (GCF), and serum have been observed 6 weeks after periodontal treatment [16]. An explanation for the different findings might be the variation in collection methods; stimulated versus unstimulated saliva, alternatively by the different use of analytical assays; Luminex versus ELISA. In the present study, chemokine concentrations were not significantly decreased as a consequence of non-surgical periodontal treatment. However, when comparing baseline concentrations with week 2 chemokine concentrations, a decrease of 32% was observed in MCP-1 concentrations, 11% in MIP-1α concentrations and 26.5% in MDC concentrations (Figure 1). The lack of statistical significance, despite the relatively big decreases observed, may be explained by the wide-range differences in inter-individual chemokine concentrations. Though a significant and steady increase in salivary concentrations of MCP-2, MDC, MIG and MIP-1α were noted from week 2 to week 12, which presumably reflects the gradual increase in local inflammation (BOP) starting in week 2 (Figure 1).

A significant correlation in baseline and week 2 concentrations for all nine chemokines was recorded. Interestingly, the number of correlating chemokines slowly decreased from week 2 until week 12 (Table 1). These results may indicate that also at the individual level the salivary chemokine concentrations were stable during the first 2 to 6 weeks after periodontal therapy. When the correlations of the different chemokine concentrations were analyzed, interactions in some chemokine groups, e.g. MCP-2/MCP-3/MCP-4, MDC/MIG/MIP-1ɑ and MCP-1/MIP-1ɑ/MIG/IP-10 (Table 2), were demonstrated. Various macrophage activation-related chemokines share receptors on target cells and activate the same pathways [29]. This information might explain the correlations of MCP-2/MCP-3 and MCP-4, as they all activate receptor CCR2, and the correlations of MIG and IP-10, as they activate receptor CXCR3. Finally, chemokine production can be induced by different inflammatory cytokines, which may also explain diversities in the expression and secretion of each chemokine [3]. In agreement with our findings, significant correlations of MCP-1, MIG and IP-10 have previously been shown during inflammation [30]. This may indicate that the salivary chemokine concentrations are prone to the interactions between various cytokines and chemokines.

Production of chemokines can be induced by bacteria. This is why significant associations between abundance of bacteria associated with periodontitis and concentrations of chemokines in inflamed tissue samples have been demonstrated [31]. Furthermore, it has been documented that stimulation of inflammatory cells with either *P. gingivalis* or bacterial LPS can cause an increase in the release of both MCP-1 and MIP-1α [2,32–34]. In line, we demonstrated a positive correlation in abundance of *P. gingivalis* with MCP-1 concentrations in saliva (Table 3). Novel findings in the present study are the correlations in abundance of *F. alocis* and *P. micra* with salivary concentrations of MDC, MIP-1α and IP-10 (Table 3). Inflammatory pathway activation depends on the activated PRR. *F. alocis* activates nucleotide-binding oligomerization domain-containing protein 1(NOD1) and *P. micra* nucleotide-binding oligomerization domain-containing 2 (NOD2) [35]. The binding to different PRRs suggests species-dependent inflammatory pathways, which may explain the variations in correlations of chemokine concentrations and bacterial abundance observed. However, the exact contribution of these bacteria, associated with periodontitis, in the inflammatory cascades remains unexplained and requires further studies.

Some limitations apply to the present study. For example, airborne diseasesand antibiotic treatment caused a drop-out of six patients, which resulted in a smaller sample size than estimated by the power calculation. Furthermore, analyses of chemokines in serum and site-specific samples (GCF or gingival biopsies) from diseased sites would have improved the understanding of the observed changes in salivary chemokine concentrations. The present use of stimulated saliva samples is a fast and easy alternative to unstimulated saliva samples, which minimizes the intra-individual variations in salivary flow rates [36]. Notably, available literature on the analyses of the salivary proteome in stimulated and unstimulated saliva samples possess conflicting results [37,38]. However, when examining the salivary microbiota comparable data have been reported in stimulated and unstimulated saliva samples [39]. Finally, it would have been interesting to include other inflammatory biomarkers such as complement proteins, as other parts of the immune system, together with chemokines, interact in the pro-inflammatory processes, and may improve the biomarker-related diagnostic potential in periodontitis patients [40].

In conclusion, treatment of periodontitis seems to influence the salivary concentrations of specific macrophage activation-related chemokines. Concentrations of these chemokines correlate with salivary abundance of particular bacteria associated with periodontitis. This indicates that chemokines in saliva reflect periodontal status and possess potential as biomarkers illustrating response to treatment.

## Data Availability

Access to data will be provided upon request (mgra@sund.ku.dk).
